# Shodagor Family Strategies: Balancing Work and Family on the Water

**DOI:** 10.1007/s12110-017-9285-z

**Published:** 2017-03-11

**Authors:** Kathrine E. Starkweather

**Affiliations:** 1 University of Missouri, 1400 University Place, Columbia, MO 65211 USA; 2 Max Planck Institute for Evolutionary Anthropology, Deutscher Platz 6, 04103 Leipzig, Germany

**Keywords:** Parental investment, Household economics, Ecology, Domestic cycle, Human behavioral ecology

## Abstract

The Shodagor of Matlab, Bangladesh, are a seminomadic community of people who live and work on small wooden boats, within the extensive system of rivers and canals that traverse the country. This unique ecology places particular constraints on family and economic life and leads to Shodagor parents employing one of four distinct strategies to balance childcare and provisioning needs. The purpose of this paper is to understand the conditions that lead a family to choose one strategy over another by testing predictions about socioecological factors that impact the sexual division of labor, including a family’s stage in the domestic cycle, aspects of the local ecology, and the availability of alloparents. Results show that although each factor has an impact on the division of labor individually, a confluence of these factors best explains within-group, between-family differences in how mothers and fathers divide subsistence and childcare labor. These factors also interact in particular ways for Shodagor families, and it appears that families choose their economic strategies based on the constellation of constraints that they face. The results of these analyses have implications for theory regarding the sexual division of labor across cultures and inform how Shodagor family economic and parenting strategies should be contextualized in future studies.

All parents face trade-offs: they must decide how much time and energy to devote to particular tasks involving direct care of children (e.g., holding, feeding, soothing) as well as provisioning. While fathers tend to allocate more time to provisioning than to childcare (Geary 2000; Marlowe 2000), mothers provide the majority of children’s direct care (Sear and Mace 2008) and are significant providers and processors of calories for their families’ diets (Kelly 1995; Lee 1968; Marlowe 2001). This is likely one reason that women’s work is almost always more compatible with childcare than men’s work (Bliege Bird and Codding 2015). Humans account for this compatibility, as well as other factors such as ecological constraints, individual condition, and the availability of alloparents, when dividing labor by sex and making decisions regarding subsistence strategies. Variation in these factors often results in predictable differences between societies in division of labor by sex (e.g., Codding et al. 2011; Jochim 1988; Marlowe 2007), though it is unknown how these factors may influence within-society differences or how they may influence the strategies employed between families to balance subsistence labor with childcare.

Labor is divided in a number of different ways, including by generation or social class, but anthropologists have focused largely on describing and explaining divisions of labor by sex because similar patterns have been observed across a number of different foraging societies—men are primarily hunters, and women, primarily gatherers (Lee and DeVore 1968; Ember 1975; Dahlberg 1981; Marlowe 2007)—and because dividing economic labor by sex in predictable ways has important implications for other aspects of human social organization, such as pair bonding (Lancaster and Lancaster 1983; Lovejoy 1981) and food sharing (e.g., Bliege Bird and Bird 1997; Hawkes 1990; Hill and Kaplan 1993; Kaplan et al. 1990).

The classic theory behind human sexual division of labor is based in formal economic models of labor specialization (Becker 1985), suggesting that females’ intrinsic advantage over males in the production and care of children leads them to specialize in economic tasks that are compatible with pregnancy and childcare. Brown (1970) further suggested that women should undertake economic activities that are low-risk, close to home, and could easily be interrupted and resumed in order to meet the needs of demanding children. Evidence to support these theories and others (see Bliege Bird 1999; Gurven and Hill 2009; Hawkes 1991; Hill 1988) is mixed. Levels of the compatibility of women’s work with childcare vary across societies (Marlowe 2007), and some studies show that women do not alter their foraging behavior significantly when encumbered by pregnancy, lactation, or the presence of young children (e.g., Goodman et al. 1985; Hurtado et al. 1992). Also, although men’s and women’s production strategies diverge from one another in a number of societies (e.g., Lee 1979), there are examples of societies where the resources pursued by men and women are similar (e.g., Hewlett 1992).

Regardless of differences in how labor is divided across societies (e.g., Bliege Bird et al. 2001; Hewlett 1992; Hurtado et al. 1992; Lee 1979), consistent trends are found in nearly all cases: men tend to pursue higher-variance resources that require taking on more risk while women tend to pursue lower-variance, lower-risk resources (Codding et al. 2011), and the work that women do is almost universally *more compatible* with childcare than is the work that men do (Bliege Bird and Codding 2015; Marlowe 2007). These trends continue in industrialized countries today with women engaging in the majority of domestic labor, whether they work outside the home or not, and men dominating labor-intensive occupations such as logging and metalworking (Bureau of Labor Statistics 2014).

Not surprisingly, there are also common cross-cultural trends in the division of childcare labor that reflect the trends in subsistence labor. Mothers provide the majority of direct care to young children (Konner 2005), whereas the amount of care provided by fathers is much more variable (e.g., Hames 1988, 1992; Hewlett 1992; Winking et al. 2009). Fathers in foraging societies spend more time caring for infants and children on average than do fathers who practice other types of subsistence (Marlowe 2000). For example, Hadza fathers held infants 5.4% of the time that they were in camp (Marlowe 1999a), though they spend time near their young children 11.6% of the day and sleep with them at night (Marlowe 1999b). Aka fathers are at the high end of the range, holding or being within an arm’s reach of their infants up to 47% of the day (Hewlett 1991). At the other end of the spectrum, fathers in pastoral societies sometimes provide 0% of direct care for infants and young children (e.g., Harkness and Super 1992; Munroe and Munroe 1992). Across all types of societies, fathers tend to interact with their children around 25–35% the amount of time that mothers do (Lamb et al. 1985). Trivers (1972) and Hrdy (1992) suggest that investment from one parent will be directly related to investment from the other parent, and Winking et al. (2009) shows that Tsimane fathers’ direct care is at least partially contingent on mothers’ care and activities.

The division of labor by sex is usually considered at the level of the community, examining why most women or men in the society undertake particular tasks (but see Bliege Bird 2007; Hewlett 1992; and Marlowe 2003 for exceptions). Marlowe (2007) pointed out that the division of labor should also be examined at the household level, suggesting that optimality of husbands’ and wives’ division of subsistence tasks may become evident only in this context. This also allows for inclusion of childcare tasks in the model, which can play an important role in determining how labor is divided (Hurtado et al. 1985). Kaplan et al. (2009) include childcare in their model of the complementarity of the division of labor, indicating that the human pair bond evolved because husbands and wives undertake productive tasks that complement each other and enable them to efficiently support the nuclear family. This model suggests that mothers’ subsistence work and direct care of children should directly complement fathers’ subsistence work and direct care of children, both in the specific tasks undertaken by each parent and in the amount of time allocated to each task (though the model also assumes self-sufficiency of the nuclear family and does not take into account sharing of childcare or food).

The Shodagor of Matlab, Bangladesh, are a seminomadic community of people who live and work on small wooden boats, surrounded by water year-round. They are one of only a few groups in the world whose people live on boats (see Ivanoff et al. 1997 and Sather 1997 for other examples). They are also culturally distinct from land-dwelling Bangladeshis (Ahmed 1962), and their lifestyle results in important ecological differences as well with respect to economic opportunities and child risk. The Shodagor face a particular set of socioecological constraints that impact the decisions they make regarding subsistence labor and childcare.

First, about half of the women in the population engage in an occupation that is entirely incompatible with childcare (Starkweather 2016). They sell household goods to women living on land, walking during most daylight hours and carrying heavy baskets on their heads. When asked, 100% of women who sell reported never having taken a dependent child with them when they sell. Other women engage in occupations that are compatible with childcare: about a third of the women fish for a living and the remainder are housewives. Almost all men in the population (90%) fish as their primary occupation (Starkweather 2016).

Possibly in response to the incompatibility of women’s selling with childcare, some Shodagor men engage in their own cross-culturally unusual behavior: they stay home for 6 months of the year as primary caregivers of their children (Fig. 1). While their wives work outside of the home for half of the year, these men are responsible for all forms of direct care of children—holding, feeding, soothing, cleaning, protecting, etc. The exact amounts of time these fathers spend in particular activities is not yet known, but in no other recorded culture do a significant portion of the population of fathers forgo work outside the home in order to stay home and care for children.

**Fig. 1:**
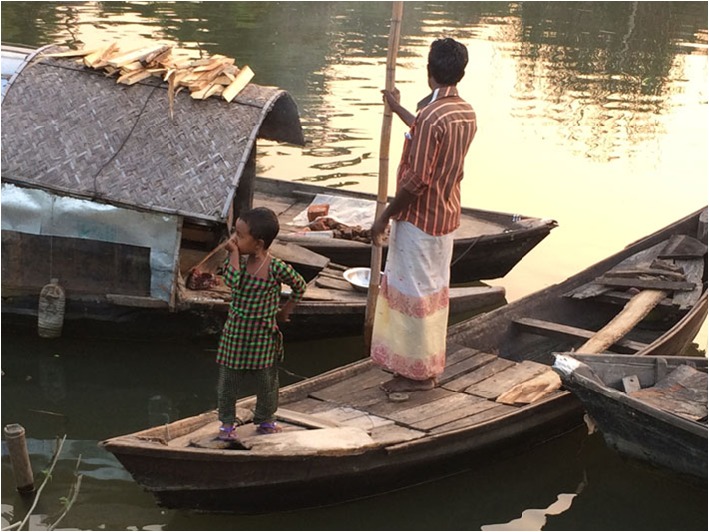
Shodagor father takes his daughter to shore on their family’s country boat while his wife works away from home selling goods. Behind the father and daughter is their family’s houseboat with firewood piled on top that the mother will use for cooking

Second, a combination of seasonality and local ecology affects work opportunities and the potential for success for both selling and fishing (for more detailed description see “Methods”). Selling primarily occurs during Bangladesh’s dry season (November to April), when roads are accessible. Proximity to a major market also affects one’s ability to sell. Women collect their goods at the market every morning prior to work, so transaction costs increase as distance from a market increases. Fishing is also affected by seasonality, with a greater number and variety of fish available during the rainy season, though the closer one lives to the Meghna River (one of the three major rivers of Bangladesh), the less the seasons affect fishing success.

Finally, the fact that Shodagor families are surrounded by water year-round impacts childcare needs. Young children in all types of environments are vulnerable to ecologically imposed dangers (e.g., Hewlett and Lamb 2005); however, life on a boat poses a constant and immediate danger. Drowning is a major cause of child death in Bangladesh for people who live on land, causing 83% of deaths for children under the age of 5 in 2003 (UNICEF 2009); it is an even greater threat for the Shodagor. This is important for two reasons. First, it requires young children to be watched diligently at all times. Shodagor children are usually considered competent swimmers around the age of 5, so younger children are constantly monitored by at least one adult (Starkweather 2016). Second, this affects the age at which older siblings and cousins, and others can serve as reliable alloparents for young children. Hames and Draper (2004) point out that whether a child can serve as a helper or not depends on suitable ecological circumstances, with semi-permanent or permanent residences and larger household sizes promoting “safe areas” conducive to allocaregiving. Reliable alloparents must be focused enough to watch children very closely and must also have the strength and swimming ability to save a child should he or she fall in the water (Starkweather 2016). These restrictions limit the overall pool of alloparents families can call on for help.

The degree to which individual families’ sets of constraints vary has resulted in families within the community employing four different strategies in order to balance economic and childcare priorities. Briefly, I label and describe these four strategies as follows (with more detailed descriptions found in “Methods”): (1) Traditional: mother stays home all year as primary caregiver for children while father works outside the home all year; (2) Split-year: mother works during the dry season while father stays home as primary caregiver, and roles reverse in the rainy season; (3) Work Together: mother and father work together all year and take the children along; and (4) Leave Kids Home: mother and father work all year (some work together, some separately) and leave children at home. The purpose of this paper is to examine between-household differences in the four strategies used to divide subsistence and childcare labor between husbands and wives. It asks the following questions: (1) Does the stage in a family’s domestic cycle affect how husband and wife divide labor? (2) How does local ecology impact division of labor between households? (3) Do alloparents affect how labor is divided? Theoretical motivations are provided and predictions are tested under the umbrella of each of these questions. Since this is the first ethnographic or empirical description of any aspect of Shodagor culture, a number of aspects of the culture are discussed in detail. Results will indicate how specific aspects of the Shodagor socioecology impact decision-making in the realms of subsistence work and childcare. Implications for the study of the division of labor cross-culturally will also be discussed.

## Does the Stage in the Family’s Domestic Cycle Affect Division of Labor?

A family’s domestic cycle refers to the normal and expected changes in age and membership a family experiences over time (Fortes 1958). For instance, in the early stages of the domestic cycle a family consists of a newly married couple. As time progresses that couple may have one baby, then potentially another. As all members of the family age, the family will go from a young couple with young, dependent children to a middle-aged couple with adolescent children, to an older couple with adult children who have families of their own. These changes can affect a number of things with regard to the division of labor. First, age and individual condition of mother and father can impact how much time they spend working and how productive they are. Kaplan et al. (2000) show that men’s rates of production in different subsistence activities across foraging societies tend to peak when men are in their mid-twenties and drop off rapidly after the age of 45 or so, depending on the activity. Women in foraging societies tend to have peak production years late in life—mostly after their reproductive careers are over. Earlier in adulthood, women are often encumbered by pregnancy and lactation. Hurtado et al. (1992) and Marlowe (2003) found that Ache and Hadza mothers (respectively) decrease or cease subsistence production during pregnancy and lactation, though for Ache mothers this decrease is less pronounced when fruits and roots are in season—resources that are less labor-intensive to acquire than others. Of Shodagor women’s three occupations, selling is most labor-intensive and the only one that is entirely incompatible with infant care. Staying home is compatible with breastfeeding and care of infants, as is fishing: women who fish often do so while breastfeeding infants. Shodagor women breastfeed exclusively for an average of 9 months and fully wean their children at 2.25 years; since the average inter-birth interval is 3.87 years, a number of premenopausal women at any given time are neither pregnant nor breastfeeding (Starkweather 2016).

- *Prediction 1: Family strategies in which mothers sell goods (split-year and leave-kids-home) are less likely to include families with pregnant or lactating mothers.*

Beyond individual condition of mother and father, the constellation of the family may have the most impact on how mother and father divide subsistence labor and childcare. As the total number of dependent children (Hurtado et al. 1992) as well as the number of weaned children (Hurtado et al. 1985) increased, Ache mothers increased both the time they spent foraging and their caloric returns. Kramer (2004) suggests that the age range of dependent children in a family will impact the number of children who are reliant on others for care and resources and the number of older siblings available to help parents meet the needs of the younger children. All of this affects the competing demands on parents to feed and provide direct care for multiple children of different ages. The implication of this is that parents with only young children are responsible for meeting all of their children’s needs—and must divide their labor accordingly. Two of the Shodagor family strategies (traditional and split-year) involve at least one parent staying home with children year-round as primary caregiver while the other parent engages in subsistence work. The other two strategies (work-together and leave-kids-home) involve both parents working away from home year-round.

- *Prediction 2: Family strategies that involve at least one parent staying home year-round (traditional and split-year) will be younger overall (or at an earlier stage in their domestic cycle) than strategies that involve both parents working year-round.*

## How Does Local Ecology Impact Division of Labor between Households?

Behavioral ecologists have shown that ecology plays a vital role in determining the subsistence strategy employed by a particular society (e.g., Hames 1979; O’Connell and Hawkes 1981; Pate 1986; Winterhalder 1981). Among other factors, the ecology determines which resources are available for human use as well as the stability of those resources. Therefore, the division of labor by sex should also be influenced by ecology (Bliege Bird 1999; Codding et al. 2011; Jochim 1988; Marlowe 2007).

Jochim (1988) suggests that optimal foraging theory, which assumes that animals will forage in a way that maximizes energy captured while minimizing time and energy expended (Stephens and Krebs 1986), can be used to explain how labor is divided by sex. However, optimal foraging theory typically focuses on an individual forager in a particular environment and ignores the potential for collecting and sharing of complementary resources. Marlowe (2007) suggests that sex-specific foraging behavior should be considered in the context of habitat variation in order to determine optimality. He hypothesizes that when husbands and wives cooperate to provide resources for a household, women will forage in a way that reflects the constraints of pregnancy, breastfeeding, and childcare, and in response men will forage optimally for foods that are not available to women. This should lead to men and women pursuing overlapping resources in less seasonal, more productive habitats, and a stricter division of labor by sex in more seasonal habitats. Results from a study using the Standard Cross-Cultural Sample support these hypotheses (Marlowe 2007). Adding risk to the model and examining the division of labor among the Ache, Martu, and Meriam in depth, Codding et al. (2011) found that habitats with high-energy, low-risk resources support a “convergent” division of labor—one in which husbands and wives work together to procure similar resources. This allows mothers and fathers to provide a reliable flow of resources to their children. Alternatively, when resources are associated with higher levels of risk, the tasks performed by men and women should diverge.

**Fig. 2:**
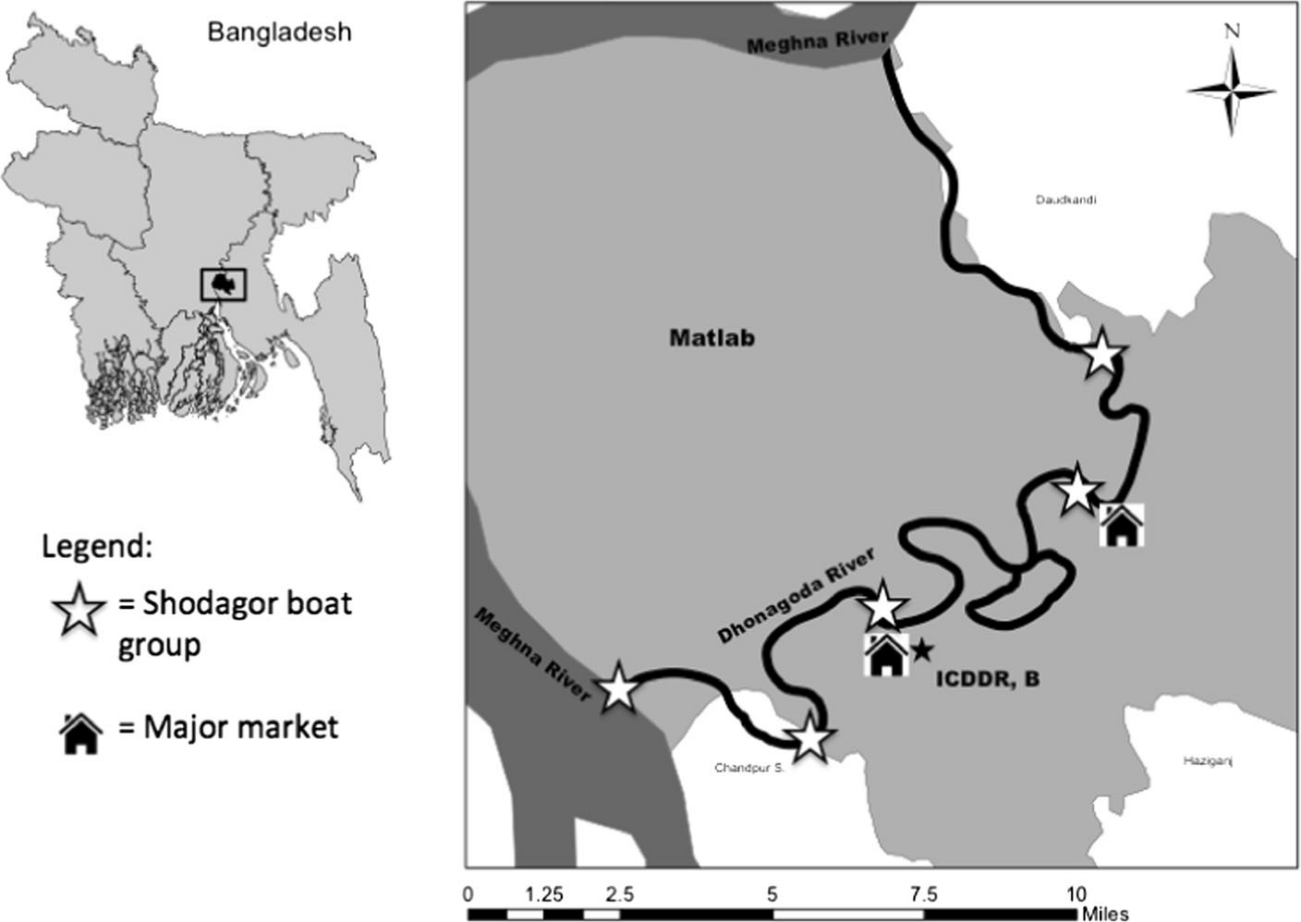
Map of Matlab, Bangladesh, with stars indicating locations of the five Shodagor bohor

Theoretically, families living in the same society should face similar ecological constraints on a broad scale—they share latitude and biome (e.g., tropical forest, desert, grassland) and are subject to the same natural occurrences. However, more specific differences between families or groups within the same society, such as distance to the nearest market town (von Rueden 2011), can impose particular ecological constraints on some families and not others. Two aspects of the local ecology in Matlab differentially affect Shodagor access to and predictability of resources: distance to the Meghna River and travel time to a major market. The five distinct Shodagor *bohor* (groups or clusters of boats) in Matlab are situated along the Donagoda River, an offshoot of the Meghna (Fig. 2). These bohor are at varying distances from the Meghna River and also from the two major market towns in Matlab. Fish is a high-energy resource that offers lowered risk of failure year-round, the closer one gets to the Meghna River. Women’s selling is a higher-risk occupation than fishing, and shorter travel time to a market enables women to sell while incurring lower opportunity costs. Distance to the Meghna and travel time to a market measure two different aspects of the ecology.

- *Prediction 3: The closer a family lives to the Meghna River, the more convergent labor will be. That is, families who engage in the work-together strategy should live closer to the Meghna than families who engage in other strategies.*
- *Prediction 4: The closer a family lives to a market, the more divergent labor will be, with women selling and men fishing. Families who engage in the split-year or leave-kids-home strategies will live closer to a market than families who engage in other strategies.*

## Do Alloparents Affect How Labor Is Divided between Husbands and Wives?

**Fig. 3:**
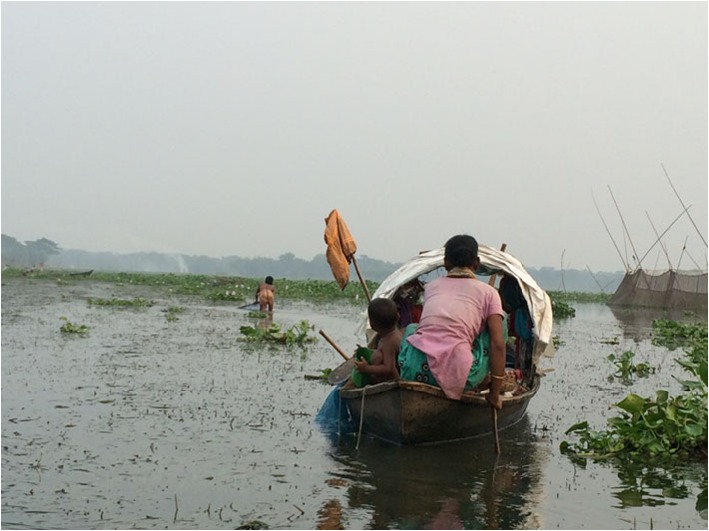
Shodagor mother rows the fishing boat while her husband fishes for small prawns in waist-deep canal water. This mother was also responsible for cooking dinner, at the other end of the boat, and caring for the two young children on the boat with her. When the father’s net is full, he and his wife will sort through the catch together

Alloparents may impact the sexual division of labor because their care frees up mothers, enabling them to spend more time away from their children (e.g., Ivey 1993, 2000). The fact that women typically engage in tasks that are more compatible with childcare than men’s tasks does not mean that children do not encumber women’s work. As Hurtado et al. (1992) showed, having a nursing infant can negatively impact a mother’s foraging production. Also, mother’s efforts directed toward parenting have been found to reduce effort that is available for subsistence activities (Hurtado et al. 1985; Ivey 1993). Alloparental care has been associated with children being weaned at earlier ages (e.g., Quinlan et al. 2003; Quinlan and Quinlan 2008) as well as mothers returning to work earlier (Quinlan et al. 2003) and spending more time working away from home (Meehan 2009). Since Shodagor mothers who sell goods spend several hours per day away from home and away from their children, they should require more alloparental help. Mothers who fish typically work with their husbands and take children with them (Fig. 3). Codding et al. (2011) suggest that a convergent division of labor in which husband and wife are performing similar subsistence tasks—and sometimes working together—may require less reliance on alloparents.

- *Prediction 5: Shodagor family strategies that involve mothers selling goods (split-year and leave-kids-home strategies) will have more alloparents available to help care for children.*
- *Prediction 6: Shodagor families engaged in the work-together strategy will have fewer alloparents available than families in other strategies.*

## Methods

### Study Population

This study focuses on the Shodagor of Matlab, Bangladesh. Matlab is a mostly rural subdistrict located within Chandpur District, approximately 59 km southeast of the capital city of Dhaka. Matlab consists of a few small towns and approximately 140 villages and is home to approximately 200,000 people (Bangladesh Bureau of Statistics 2010; International Centre for Diarrheal Disease Research, Bangladesh [ICDDR,B] 2014). The Donagoda River cuts through the middle of Matlab and flows into the Meghna at the southernmost tip of Matlab’s landmass, just east of where the Meghna and Padma rivers meet. Shodagor people live throughout Bangladesh, though the exact locations and numbers are currently unknown. No previous research has been published on the Shodagor; all ethnographic data included here comes from observations, informal conversations, AND qualitative and quantitative interviews I conducted with the help of two Bangladeshi research assistants during 2011 and 2014.

The Shodagor are distinct from land-dwelling, village Bangladeshis (whom the Shodagor call *grihosto*) in a number of ways, including cultural aspects such as religion, political organization, subsistence strategies, and kinship structure (see Novak 1993 for a detailed description of village Bangladeshi culture). The Shodagor speak a distinct dialect of Bengali as well as a Shodagor-specific language. They are Muslim but are often described by other Bangladeshis as “Muslim in name only” because their religious observances and ceremonies more closely resemble ancient Hindu traditions than Muslim practices. Shodagor also have an animist belief system, recognizing and worshiping the major rivers of the region during semi-annual ceremonies.

Marriage is primarily monogamous with mild levels of polygyny. Divorce occurs, but usually early in a marriage before the birth of the first child. The primary reason for dissolution of marriage is death of a spouse. Most marriages are arranged by parents or relatives, though love marriage is not atypical, and most are endogamous within the Shodagor subculture. Marriage payments among the Shodagor have recently begun to shift from a primarily brideprice system toward dowry, a shift that village Bangladeshis underwent approximately 50 years ago (Amin and Cain 1997). Kinship is recognized bilaterally: inheritance of identity is traced through the father, whereas property is inherited from both mother and father. As is common among nomadic and semi-nomadic groups (Ember 1975), Shodagor postmarital residence patterns are multilocal. My data show that almost half (49%) of ever-married individuals in Matlab lived patrilocally after marriage, whereas 21% lived matrilocally, 16% lived bilocally (near both husband’s and wife’s families), and 14% lived neolocally. While there are always exceptions to the rule, village Bangladeshis, in contrast, have strong and long-standing patrilineal, patrilocal traditions (Aziz 1979).

### Bohor Structure

Traditionally, all Shodagor were seminomadic, moving two or three times per year to different locations throughout Bangladesh. Currently approximately half of all Shodagor families in Matlab move an average of two times each year—some to locations throughout the country and some between *bohor* (distinct groups or clusters of boats) in Matlab. Other Shodagor families in Matlab move very infrequently or not at all. Unless they are moving between locations, families always situate their houseboats within preexisting bohor—houseboats never reside permanently outside of a bohor. Smaller boats are used as the primary mode of transportation for fishing and for traveling between the houseboat and the land. Families choose to live in particular bohor for a number of reasons, including wanting to be close to relatives, for work opportunities, and because older generations lived in the same bohor.

The five Shodagor bohor in Matlab range from 8 to 18 households each, with an average of 15 households. The largest bohor included in this study is made up of 32 Shodagor families who have moved onto the land within the past 5 years and live in makeshift houses on very small pieces of land. These families are heavily integrated with those living on boats through kin and cultural ties. Other Shodagor families in Matlab who have lived in houses that are similar to land-dwelling Bangladeshis’ for 10 or more years were not included in this study.

Shodagor political organization is largely status egalitarian, though each bohor has a recognized *shordar* (headman). Shordars obtain their position either through primogeniture, with the oldest son inheriting leadership status from his father, or through an election held among the other shordars if (*a*) no older son or eligible relative of the previous shordar is available or (*b*) a new bohor is formed. It is agreed upon by most Shodagor that families who live in houses have slightly higher status than those who live on boats and that those who have lived in houses the longest have the highest status within the community. The Shodagor are also relatively gender egalitarian, with women in many families playing a role in decision-making within the family and within Shodagor society and most women moving freely in public and private spaces. The autonomy of Shodagor women is strikingly different from that of village women, for whom purdah is a common practice, women’s sexual reputations are closely guarded, and work outside the home is uncommon (Amin 1997).

### Domestic Cycle

Shodagor boys and girls live at home with their parents in nuclear family households throughout childhood and until they get married. Based on data collected by the author (using methods that will be described below), for Shodagor men, first marriage occurs at the age of 23 years, on average, whereas women marry at the age of 16.5 years on average. Men marry an average of 1.4 times in their lives and women marry an average of 1.14 times in their lives. After marriage, the new couple moves into their own boat, which they often pay for themselves, and form a new household. Men average 24.7 years of age when their first child is born and women average 17.8 years of age at first birth. Women who have likely completed fertility (those 45 years or older) have an average of 5.7 children; however, preliminary analyses of birth spacing data suggest that total fertility for women in more recent generations will be lower.

### Economy: Men’s Work

Shodagor men primarily work as fishermen, with 90% of the men in Matlab fishing for at least some portion of the year. Men sell their catch in the markets in exchange for money, though almost all practice subsistence fishing as well. Some men also engage in day labor (11%), selling household goods (7%), and other types of work (2%) throughout the year, with 18% of men reporting more than one occupation. Regardless of occupation, neither fish nor cash are shared extensively outside of nuclear families.

### Economy: Women’s Work

Almost half (44%) of Shodagor women work at least half of the year selling goods in markets and door-to-door in villages. Some women also fish with their husbands (31%) for all or part of the year and others are primarily housewives (34%) for at least a portion of the year. These economic roles differ dramatically from those of the landed Bangladeshis, with men heavily engaged in agriculture and wage labor and women rarely working outside the home (Amin 1998).

### Bangladeshi Ecology

The ecology of Bangladesh plays an important role in Shodagor lifestyle. Every year, for approximately half of the year, large portions of the entire country of Bangladesh are covered in water, as a result of monsoon rains and Himalayan snowmelt (Hofer and Messerli 1997). The Shodagor refer to this half of the year, which typically begins in June, as the “rainy season.” During June, July, and August, monsoon rains come regularly, but for the Shodagor, the season usually extends through the end of October, when the waters have receded to the point that roads are dry and accessible. The other months of the year are referred to as the “dry season,” when water levels in the country’s rivers and canals decrease and land is visible in places that previously were completely submerged.

Although the actual living conditions of most Shodagor do not change drastically between the seasons (they continue to live on boats on the water all year), what do change are the economic opportunities. During the dry season, fishing prospects vary across Bangladesh, but as water levels rise, fish become abundant throughout the country, with the availability of larger amounts and different types of fish making fishing a particularly profitable venture. During this half of the year, nearly all Shodagor men and some women fish every day. Selling goods is nearly impossible and highly unprofitable for women during the rainy season because many of the roads in the rural parts of the country are submerged in water. This makes transportation and access to potential customers very difficult. The dry season brings access to roads, making it a profitable time for women to sell goods.

### Local Ecology

Two aspects of the specific ecology of Matlab are important for Shodagor. First, the distance a family lives from a market town will determine the ease with which women can obtain goods to sell. Women do not store the goods they sell in their own homes; instead they are held by a *mahajan* (middleman) in a shop in the nearest major market. Each selling day, women travel first to the market to collect their basket of goods, then out to the countryside to sell. Before returning home, women deposit their goods in the shop for the night and settle accounts with the mahajan. There are two major market towns in Matlab and two of the five bohor are located within a 5- or 10-min walk from those towns, whereas the other three bohor are located an hour or more away. Distance to market is less important for the success of men’s fishing. Men do sell most of their fish in markets at the end of each day, but this can be done at major and minor markets. Minor markets are located all over the countryside, and all five bohor in Matlab live within walking distance of a minor market.

The second aspect of the Matlab ecology that is relevant to the Shodagor is the distance a family lives from the confluence of the Donagoda and the Meghna, as this will determine fishing opportunities throughout the year. The Donagoda is the main river that runs from north to south through the middle of Matlab. The average width of the Meghna is approximately 5 km, which is nearly equivalent to the widest point of the Mississippi River, and at its widest point the Meghna is nearly 10 km from shore to shore during the dry season and even larger during the rainy season. At the mouth of the Donagoda, the river is wide and deep and there is an abundance of fish, in both number and variety, year-round. Upstream on the Donagoda, especially during the dry season, water levels are lower and numbers and varieties of fish diminish. Shodagor who live closer to the Meghna will have year-round fishing opportunities that are potentially profitable, whereas those who live farther away will find fishing most profitable during the rainy season and less profitable during the dry season.

### Data Collection

The following study is based on interview data that were collected over the course of 12 months of fieldwork during 2011 and 2014. All adult Shodagor living in Matlab were eligible to participate in the interview portions of this study. An individual was considered an adult if (*a*) he or she was aged 18 or older at the time of the interview and (*b*) had ever been married. Among the Shodagor, an individual is considered to be a social adult when he or she gets married, regardless of age. However, Institutional Review Boards in the US consider an individual to be an adult at the age of 18, regardless of marital status. The only married individuals under the age of 18 were female; therefore, when interviewing married individuals under the age of 18, consent was obtained from both the woman being interviewed and her husband or a parent, and the husband or parent was also present during the interview. Individuals were excluded from this study if they declined to participate, if they were away from home during the study period, or if they had health conditions that precluded them from participating.

After obtaining informed consent from all participants, quantitative survey interviews were conducted in two rounds. The first round was conducted between May and July 2014. It had more than 232 questions, lasted between 45 and 90 min, and focused on demographic information and family histories, basic economic data (such as income and occupation data), indirect parental investment data, and family and women’s health histories. Of the 172 total adult Shodagor in Matlab, 71 men and 87 women participated in this interview (*N* = 158), accounting for an almost complete population sample. Fourteen adults either declined to participate, for reasons including health issues, or were away from home every time interviews were conducted. The second survey was conducted in September and October 2014 and asked questions about specifics of economic practices and income, parental investment, and collected household inventories. A smaller sample of 34 men and 41 women participated in this survey. For logistical reasons, men and women were asked to participate in this second survey if they lived in one of the three bohor located closest to the Meghna River.

The current study encompasses 64 Shodagor families representing all five bohor in Matlab. Families were included if they had dependent children at the time of the study, if both parents lived in the household (boat or house), and if data were available on income and occupation over the preceding year. Families were excluded if they did not meet the above criteria. For example, one family was excluded from the analysis because the parents were divorced, and another family was excluded because the mother had mental health problems that disabled her from both work and childcare activities. All data in this study are cross-sectional.

### Variables

The outcome variable for this study is *family strategy*. Families were coded with a particular strategy for the preceding year based on reports of seasonal employment and income that were given as part of the two surveys. In the first survey, respondents were asked about income earned during the previous rainy season and dry season as well as primary and secondary occupations. Seasonal income reports from the previous year—whether or not a particular man or woman earned income during each season—were used to create the family strategy categories (amount of income was not used for this purpose). Individuals were also asked to report whether or not they worked together with their spouse for half of the year or all of the year. In the second survey, individuals were asked what type of work they do in each season (e.g., fishing, selling goods, childcare, does not work) and who cares for their children while they work. These data points, along with observation data regarding whether children go fishing with their parents or are left home, were used to determine the particular strategy each family practices and to code them accordingly. This is a categorical variable with four distinct family strategies: traditional (*N* = 17), split-year (*N* = 17), work together (*N* = 12), and leave kids home (*N* = 18).

Families that employ the traditional strategy most closely resemble the land-dwelling Bangladeshi families in terms of economic and parenting behaviors. In these families, the father works all year while the mother stays home all year as primary caregiver for their children.

Some Shodagor parents split the year between economic and parenting pursuits, with mother and father each working for approximately half the year. In these families, fathers typically fish during the rainy season while mothers stay home with the children, and mothers work during the dry season, selling goods door-to-door, while fathers stay home with the children.

Other Shodagor parents work together all year and take the children along with them. The primary occupation of both mother and father in these families is fishing, so each plays an important role on the boat, with the father typically checking the hooks or nets while the mother rows the boat and sorts the fish. Children who are old enough to help in some way often do, while younger children play on their own. It is unknown who provides the most care for young children during the workday, but some families begin taking their children to work with them as soon as the mother is sufficiently recovered from childbirth. Infants will then often sleep on their mothers’ laps, waking to nurse while mother is rowing the boat.

Finally, in some Shodagor families, both mother and father work all year, whether together or separately, but leave their children at home. In these cases, an alloparent may look after the children or, if the children are considered old enough, they may stay on their own all day without any supervision.

Predictor variables (listed in Tables 1 and 2) were selected specifically to test the predictions outlined above. The number of predictor variables in the multivariate model was limited to six based on an a priori power analysis, and the variables were selected based on bivariate results and theoretical relevance. *Mother’s age* and *child under 5 (dummy)* were used in the multivariate model as variables representative of a family’s stage in the domestic cycle. Mother’s age was chosen rather than father’s age and average age of children because (1) all three are highly collinear (mother’s age and father’s age: *r*_75_ = 0.90, *p* < 0.001; mother’s age and average age of children: *r*_78_ = 0.93, *p* < 0.001; father’s age and average age of children: *r*_69_ = 0.88, *p* < 0.001) and (2) mother’s age is a biologically limiting factor in the family’s ability to reproduce. Whether or not a family has a child under the age of 5 was chosen because it represents an important constraint on families’ childcare needs. *Time to market (dummy)* and *distance to the Meghna (in km)* are ecological variables that each measure distinct aspects of a family’s ecology, as explained above. Of course in a seminomadic culture, “local” ecology is relative to the location of a family at any given point in time. In this study, both measures of ecology were collected at the time of the initial interview and at the time that the data regarding family strategy was collected. Time-to-market represents the ease with which women can sell goods as one of their primary occupations. This was first recorded as a continuous variable but was recoded into a binary variable in order to distinguish between families who live within 10 min of a market (coded as 0) and those who live more than 45 min away (coded as 1) since no families are located between 10 and 45 min from a market. The distance to the Meghna River indicates the potential profitability of fishing during different seasons. *Number of available alloparents* measures the number of individuals reported in the survey as available allocaregivers who also live in the same bohor as each family. Finally, *household income* is included in the model as a control variable to ensure that income is not accounting for the majority of variability between strategies.

**Table 1:** Summary statistics (*N*) and chi-square results for categorical variables, comparing families across four strategies

Variable	Traditional	Split year	Work together	Leave kids home	χ^2^
Outcome variable					
Family strategy	19	17	13	18	
Domestic cycle					
Mother pregnant/Breastfeeding					
Yes	4	2	2	2	0.904
No	15	15	11	16	
Child under age 5					
Yes	14	14	7	4	15.629**
No	5	3	6	14	
Dowry					
Yes	4	4	5	3	2.121
No	15	13	8	15	
Ecology					
Time to market					
< 10 min	13	15	1	15	25.778***
> 10 min	6	2	12	3	
Alloparents					
Available alloparent					
Yes	7	12	4	7	6.156
No	12	5	9	10	
Paternal grandfather in group					
Yes	4	4	4	1	3.499
No	15	13	9	17	
Paternal grandmother in group					
Yes	6	7	5	4	1.656
No	13	10	8	14	
Maternal grandfather in group					
Yes	6	5	4	1	4.570
No	13	12	9	17	
Maternal grandmother in group					
Yes	6	7	6	3	3.729
No	13	10	7	15	
Child over age 10					
Yes	4	8	5	12	8.056*
No	15	9	8	6	

Statistical significance is represented as follows: *** *p* < .001, ** *p* < .01, * *p* < .05, **†** *p* < .10

**Table 2:** Summary statistics (mean ± SD) and ANOVA analyses for continuous variables

Variable	Traditional	Split year	Work together	Leave kids home	*F*
Domestic cycle					
Mother’s age	25.68 ± 9.20	28.35 ± 9.25	30.46 ± 12.50	35.67 ± 10.38	3.122*
Father’s age	34.56 ± 11.07	38.59 ± 13.62	35.00 ± 14.76	45.88 ± 11.87	2.667†
Average age of children	6.05 ± 6.74	7.85 ± 6.31	10.58 ± 9.16	14.07 ± 7.07	4.212**
Number of children	2.16 ± 2.19	2.88 ± 2.20	3.15 ± 1.68	3.39 ± 1.69	1.324
Dowry payment (ln)	2.03 ± 4.06	2.19 ± 4.09	3.24 ± 4.30	1.54 ± 3.54	0.474
Ecology					
Time to market (min)	35.00 ± 46.10	14.12 ± 28.95	62.69 ± 28.40	14.17 ± 22.90	6.920***
Distance to meghna (km)	8.48 ± 5.90	9.42 ± 4.34	2.94 ± 2.68	6.61 ± 2.09	6.882***
Alloparents					
*N* Available	0.42 ± 0.61	1.06 ± 0.83	0.31 ± 0.48	0.41 ± 0.51	4.899**
Control					
Household income (ln)	11.41 ± 0.72	11.92 ± 0.79	11.78 ± 0.66	11.49 ± 0.69	1.817

Statistical significance is represented as follows: *** *p* < .001, ** *p* < .01, * *p* < .05, **†** *p* < .10

### Analyses

The relationships between family strategy and categorical predictor variables were examined using a chi-square test. If an overall effect was found, follow-up pairwise comparisons were performed in order to determine the significance of mean differences between each pair of conditions. For continuous predictor variables, the relationships between the outcome and the predictors were examined using an ANOVA test in order to determine whether or not there are significant mean differences among families employing different strategies. Independent-samples *t*-tests were then run in order to determine between-strategy differences.

Next, given that *family strategy* is a categorical outcome variable, a multinomial logistic regression was conducted to test the above predictions and look for factors that distinguish one strategy from another. Variables included in the model were based on bivariate results and an a priori power analysis. When conducting a multinomial logistic regression, a reference category is chosen and the maximum likelihood of each predictor variable for all other categories is compared with the reference. The goal of this analysis is to determine how each category compares with the others, and there is no empirical or theoretical reason for justifying using one category or family strategy as the baseline. Therefore, the regression was conducted four times, each time using a different family strategy as the reference category in order to test predictions and fully understand which of these six independent variables significantly distinguish between family strategies.

## Results

### Does the Stage in the Domestic Cycle Affect Division of Labor?

Bivariate results did not support the first prediction, which states that family strategies in which mothers sell goods (split-year and leave-kids-home) should be less likely than other families to have a mother who is pregnant or breastfeeding. The chi-square test showed that families in all strategies are equally likely to have a pregnant or breastfeeding mother (Table 1). Based on this result as well as the fact that the variable *pregnant or breastfeeding mother (dummy)* did not contribute significantly to the multivariate model, it was not included in the final model.

Bivariate analyses show mixed results regarding the second prediction, which states that families practicing traditional and split-year strategies should be younger than those practicing work-together and leave-kids-home strategies. The chi-square test found that families in traditional and split-year strategies were more likely to have a child under the age of 5 than families in the leave-kids-home strategy; however, families who work together are as likely to have a child under 5 as they are not to, and significantly equally likely as families in all other strategies (Table 1). ANOVA results, confirmed using *t*-tests, show that mothers (*t*_35_ = 3.101, *p* = 0.004), fathers (*t*_32_ = 2.877, *p* = 0.007), and children (*t*_35_ = 3.533, *p* = 0.001) in families who practice the traditional strategy and mothers (*t*_33_ = 2.196, *p* = 0.035) and children (*t*_33_ = 2.733, *p* = 0.010) in families who practice the split-year strategy are significantly younger than those who practice the leave-kids-home strategy. However, fathers in the split-year strategy are not significantly younger than those in the leave-kids-home strategy, and mothers, fathers, and children in both traditional and split-year strategies are not significantly younger than those in the work-together strategy (Table 2).

Results from the multinomial logistic regression show that when controlling for ecology, number of available alloparents, and household income, mother’s age was not significantly different between any of the four strategies, and families in the traditional and split-year strategies were only marginally significantly more likely than those in the leave-kids-home strategy to have a child under the age of 5 and equally as likely as families who work together (Tables 3 and 4).

**Table 3:** Multinomial logistic regression outcomes (χ^2^ = 68.75, Pseudo R^2^ = 0.66, *N* = 63), compared with traditional family strategy

Variable	Odds ratio (95% CI)		
	Split year	Work together	Leave kids home
Domestic cycle			
Mother’s age	1.063 (0.94, 1.21)	0.989 (0.86, 1.14)	1.062 (0.96, 1.18)
Child under 5 (Dummy)	1.461 (0.13, 17.14)	0.388 (0.03, 5.31)	0.122 (0.01, 1.27) †
Ecology			
Time to market (Dummy)	0.385 (0.05, 2.89)	20.645 (1.20, 355.11)*	0.082 (0.01, 1.33) †
Distance to meghna (Km)	1.279 (1.01, 1.62)*	0.833 (0.63, 1.11)	0.808 (0.58, 1.14)
Alloparents			
*N* Available	5.909 (1.22, 28.52)*	0.175 (0.02, 1.68)	2.808 (0.53, 14.91)
Control			
Household income (ln)	3.514 (0.96, 12.81)†	2.201 (0.37, 12.96)	1.293 (0.39, 4.29)

Statistical significance is represented as follows: *** *p* < .001, ** *p* < .01, * *p* < .05, **†** *p* < .10

**Table 4:** Multinomial logistic regression outcomes, compared with split-year family strategy

Variable	Odds ratio (95% CI)		
	Traditional	Work together	Leave kids home
Domestic cycle			
Mother’s Age	0.941 (0.83, 1.06)	0.931 (0.79, 1.10)	0.999 (0.89, 1.12)
Child under 5 (Dummy)	0.684 (0.06, 8.03)	0.266 (0.01, 6.26)	0.083 (0.01, 1.32) †
Ecology			
Time to market (Dummy)	2.596 (0.35, 19.46)	53.596(2.12, 1355.53)*	0.214 (0.01, 4.84)
Distance to meghna (Km)	0.782 (0.62, 0.99)*	0.651 (0.46, 0.92)*	0.632 (0.44, 0.91)*
Alloparents			
*N* Available	0.169 (0.04, 0.82)*	0.030 (0.01, 0.37)**	0.475 (0.09, 2.43)
Control			
Household income (ln)	0.285 (0.08, 1.04)†	0.626 (0.09, 4.47)	0.368 (0.09, 1.40)

Statistical significance is represented as follows: *** *p* < .001, ** *p* < .01, * *p* < .05, **†** *p* < .10

### How Does Local Ecology Impact Division of Labor between Households?

**Fig. 4:**
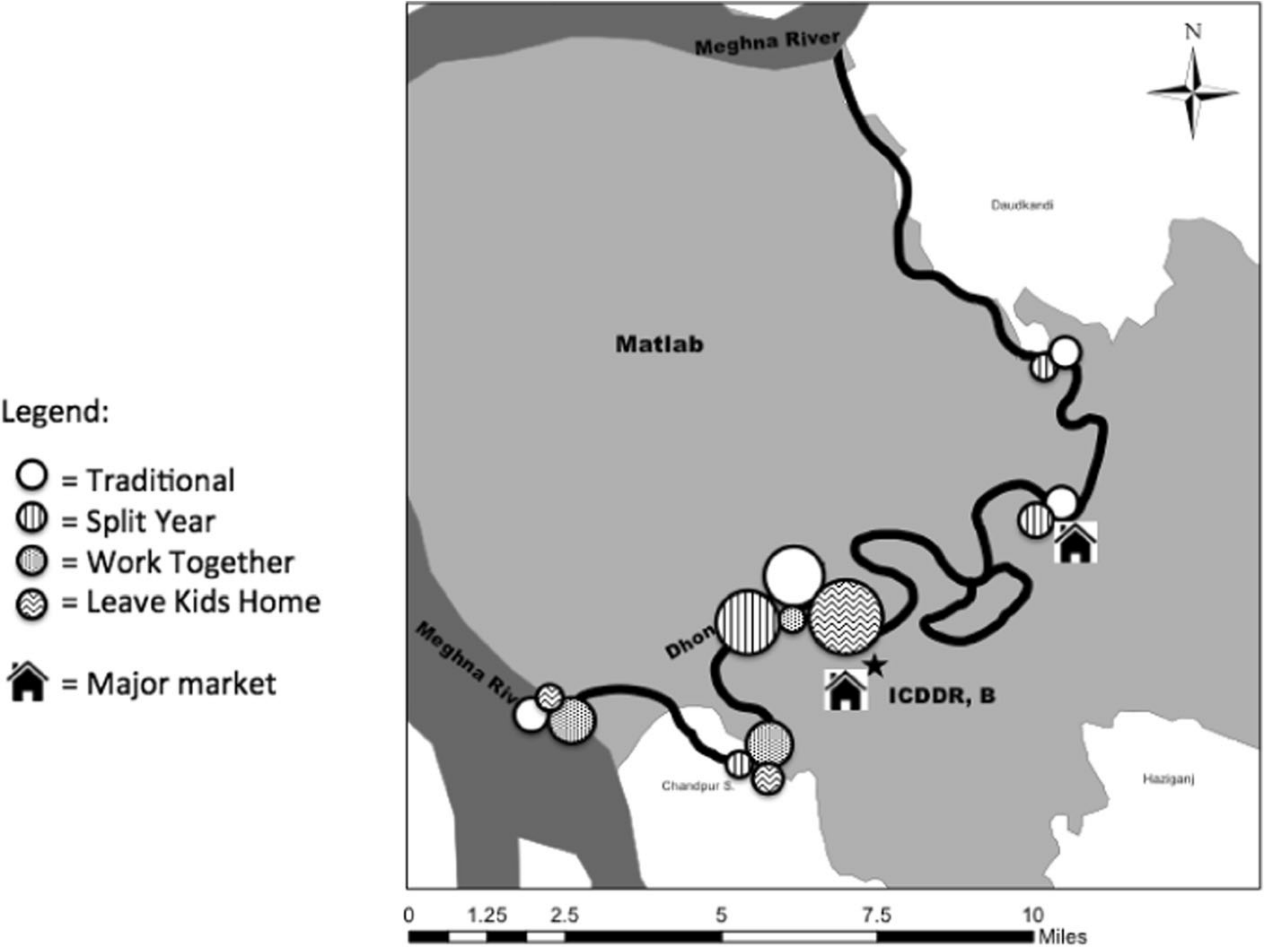
Map of family strategy by bohor location

Figure 4 visualizes the proportion of families in each bohor who employ each strategy. Prediction 3 states that families who work together should live closer to the Meghna River than families in other strategies. Bivariate results clearly support this prediction, with work-together families living significantly closer to the river than traditional families (*t*_30_ = 3.158, *p* = 0.004), split-year families (*t*_28_ = 4.724, *p* < 0.001), and leave-kids-home families (*t*_29_ = 4.276, *p* < 0.001) (Table 2). Multinomial logistic regression results (Table 5) only partially support this prediction. Families who work together are more likely to live closer to the Meghna than families who employ the split-year strategy, when controlling for domestic cycle variables, number of available alloparents, and household income. However, families in the split-year strategy are also more likely to live farther from the Meghna than traditional or leave-kids-home families (Table 4).

**Table 5:** Multinomial logistic regression outcomes, compared with work-together family strategy

Variable	Odds ratio (95% CI)		
	Traditional	Split year	Leave kids home
Domestic cycle			
Mother’s age	1.011 (0.88, 1.17)	1.075 (0.91, 1.27)	1.074 (0.93, 1.24)
Child under 5 (Dummy)	2.574 (0.19, 35.22)	3.762 (0.16, 88.55)	0.313 (0.02, 5.36)
Ecology			
Time to market (Dummy)	0.048 (0.01, 0.83)*	0.019 (0.01, 0.47)*	0.004 (0.00, 0.15)**
Distance to meghna (Km)	1.200 (0.90, 1.60)	1.535 (1.09, 2.17)*	0.970 (0.64, 1.46)
Alloparents			
*N* Available	5.703 (0.59, 54.75)	33.700 (2.66, 424.43)**	16.014 (1.39, 184.27)*
Control			
Household income (ln)	0.454 (0.08, 2.67)	1.596 (0.22, 11.39)	0.587 (0.10, 3.36)

Statistical significance is represented as follows: *** *p* < .001, ** *p* < .01, * *p* < .05, **†** *p* < .10

Bivariate and multivariate results only partially support Prediction 4, which suggests that families in the split-year and leave-kids-home strategies will live closer to a major market than families in the other strategies. An ANOVA showed that although split-year (*t*_28_ = 4.591, *p* < 0.001) and leave-kids-home (*t*_29_ = 5.265, *p* < 0.001) families live closer to a market than work-together families, neither live significantly closer to a market than traditional families (Table 2). Chi-square results (Table 1) and multinomial logistic regression results (Table 3, 4, 5 and 6) support this pattern. Split-year and leave-kids-home families, along with traditional families, are more likely to live less than 10 min from a major market than work-together families. Leave-kids-home families are also more likely to live less than 10 min from a major market than traditional families.

**Table 6:** Multinomial logistic regression outcomes, compared to leave kids home family strategy

Variable	Odds ratio (95% CI)		
	Traditional	Split year	Work together
Domestic cycle			
Mother’s age	0.941 (0.85, 1.05)	1.010 (0.90, 1.12)	0.931 (0.81, 1.07)
Child under 5 (Dummy)	8.222 (0.79, 85.74) †	12.014 (0.76, 189.98) †	3.194 (0.19, 54.69)
Ecology			
Time to market (Dummy)	12.125 (0.75, 196.19) †	4.671 (0.21, 105.55)	250.322 (6.85, 9152.24)**
Distance to meghna (Km)	1.237 (0.88, 1.74)	1.582 (1.10, 2.28)*	1.031 (0.68, 1.56)
Alloparents			
*N* Available	0.356 (0.07, 1.89)	2.104 (0.41, 10.74)	0.062 (0.01, 0.72)*
Control			
Household income (ln)	0.774 (0.23, 2.56)	2.719 (0.72, 10.34)	1.703 (0.30, 9.23)

Statistical significance is represented as follows: *** *p* < .001, ** *p* < .01, * *p* < .05, **†** *p* < .10

### Do Alloparents Affect How Labor Is Divided between Husbands and Wives?

Prediction 5, which states that split-year and leave-kids-home families will have more alloparents available than traditional or work-together families, was only partially supported. A chi-square test shows no significant differences between strategies in whether or not a family has any alloparent available (Table 1). ANOVA results show that split-year families have more alloparents available than families in all other strategies—traditional (*t*_34_ = 2.657, *p* = 0.012), work-together (*t*_28_ = 2.913, *p* = 0.007), and leave-kids-home (*t*_32_ = 2.750, *p* = 0.010)—and no other strategies are significantly different from one another (Table 2). Multinomial logistic regression results show that when controlling for all other variables in the model, split-year families have significantly more alloparents available than traditional and work-together families, but not more than leave-kids-home families (Table 4). Also, leave-kids-home families have significantly more alloparents available than work-together families (Table 5). These findings also partially support Prediction 6, which suggests that work-together families will have fewer alloparents available than families in any other strategy.

### Strategy-by-Strategy Breakdown

After controlling for the other variables in the multinomial logistic regression model, families employing the traditional strategy are more likely to have a child under the age of 5 and live farther from a market than leave-kids-home families and closer to a market than work-together families. They also live closer to the Meghna River, have fewer alloparents available, and a lower household income than split-year families.

Families employing the split-year strategy are more likely to have a child under the age of 5 in the household than the leave-kids-home families. They live closer to a market than work-together families, have more alloparents available than traditional and work-together families, and have a higher household income than traditional families. These families live farther from the Meghna River than families in all other strategies.

Families employing the work-together strategy live farther from a market than families in all other strategies. They also live closer to the Meghna than split-year families and have fewer alloparents available than split-year or leave-kids-home families.

Leave-kids-home families are less likely to have a child under the age of 5 in the household than the traditional or split-year families. They live closer to a major market than traditional or work-together families, live farther from the Meghna than split-year families, and have more alloparents available than work-together families.

## Discussion

Though several models have been examined, no single model effectively explains the division of labor between men and women in a society or a household (Bliege Bird and Codding 2015). These results indicate that a family’s stage in the domestic cycle, their local ecology, and the availability of alloparents will affect how they divide subsistence and childcare labor at the household level. These factors also interact in particular ways for Shodagor families, and it appears that families choose their economic strategies based on the constellation of constraints that they face. The results of these analyses have implications for theory regarding the sexual division of labor across cultures and inform how Shodagor family economic and parenting strategies should be contextualized in future studies.

### Stage in Domestic Cycle Predicts Parents’ Year-Round Proximity to Children

A number of factors contribute to the stage of the domestic cycle a family inhabits at any given moment, including parents’ ages, age of children (i.e., age distribution, average age of all children, age of youngest child), and mother’s breastfeeding status. Theory (e.g., Brown 1970; Kaplan et al. 2009) and ethnographic evidence (e.g., Hurtado et al. 1992; Marlowe 2003) suggest that mothers who are pregnant or breastfeeding should engage in work that is most compatible with their physical limitations. However, Shodagor mothers who were either pregnant or exclusively breastfeeding at the time of this study were no more likely to engage in one family strategy—or one occupation (χ^2^_2_ = 0.784, *p* = 0.676)—over another. One possible explanation for this is that only 10 women in the society were either pregnant or exclusively breastfeeding at the time the survey was conducted. More data may reveal differences across strategies that do not currently show up.

Clear evidence for the impact of the family’s stage in the domestic cycle on how they divide labor is found when examining age variables. For the Ache (Hurtado et al. 1992) and the Maya (Kramer 2004), the stage in a family’s domestic cycle affects parents’ ability to spend time working away from home and away from children. On average, Shodagor traditional and split-year families are younger than leave-kids-home families, in which both parents work away from home and their children. What seems to be the most important factor, though, is whether or not a family has a child under the age of 5. Even when controlling for mother’s age, families who have at least one child under the age of 5 are significantly more likely to have either the mother or father stay home with the child(ren), whereas those without a child under the age of 5 are likely to have both mother and father leaving the children to work away from home.

Drowning is a primary cause of death for children under the age of 5 in Bangladesh (UNICEF 2009; ICDDR,B 2014) and is of major concern for Shodagor parents. One prevention strategy is to have a responsible and able-bodied caregiver with children at all times. The risk of drowning is likely the main reason parents with young children undertake a strategy that involves a parent being the primary caregiver and simultaneously forgoing an opportunity to work and earn money. Once children are confident swimmers, it seems that most mothers and fathers will choose a strategy that allows both adults in the family to make money.

Contrary to expectations, parents who work together all year and take kids along with them are just as likely to have a child under the age of 5 as families in all of the other strategies. Work-together families may be the most flexible in terms of childcare, given that both parents are present on the boat and at least one can easily be within arm’s length of young children at all times. Mothers who fish can carry infants on their laps while they row the boat or sort through fish. Families who take older children fishing with them usually put those children to work on the boat. Therefore, the family’s stage in their domestic cycle—and specifically whether they have a child under the age of 5—distinguishes between families in which one parent stays home with children year-round and families in which children are left at home without a parent, but does not distinguish work-together families from others.

### Local Ecology Determines Subsistence Opportunities

In a cross-cultural study of foragers, Marlowe (2007) found that the types of resources men and women pursue diverge from one another in environments where resources are affected by seasonality. Codding et al. (2011) suggest that the division of labor should diverge when resources that provide high returns can only be acquired at high levels of risk. For the Shodagor, the circumstances that lead to a divergent division of labor in both models overlap. Families who live closer to the Meghna River are less affected by seasonality, as fish are abundant year-round, whereas for those who live farther from the Meghna, fish are most consistently available during the rainy season. Proximity to a major market provides women with opportunities to sell goods, an occupation which is also affected by changing seasons and is possible for most women only during the dry season.

In support of both Marlowe (2007) and Codding et al. (2011), proximity to a major market is associated with a divergent division of labor: families who practice split-year and leave-kids-home strategies live closer to a major market than families who work together. Women in these two strategies sell goods for at least half of the year and men fish. Women’s ability to sell, in particular, is impacted by seasonality and outcomes are high-risk, high-reward. Families employing the traditional strategy also live closer to a major market than families who work together, and the traditional strategy also represents a divergent division of labor, with men working outside the home year-round and women staying home, managing the household and caring for children. There is no reason to believe that either men or women in this strategy are affected by seasonality, nor is there evidence to suggest that men are undertaking particularly high-risk occupations. Most men whose wives stay home either fish year-round or work in shops or in the market, for which proximity to a major market may be providing alternative work opportunities.

Also in support of Marlowe (2007) and Codding et al. (2011), proximity to the Meghna River is associated with a convergent division of labor: families who work together, in which husband and wife fish together year-round, live closer to the Meghna than split-year families. However, traditional families and leave-kids-home families live closer to the Meghna than split-year families, too. Leave-kids-home families provide an interesting case because whereas the work men and women do is divergent for half of the year (with women selling and men fishing), most families engage in convergent labor during the other half of the year (with men and women fishing together). It makes sense for these families to live close to the Meghna and close to a major market.

A family’s time to market and distance to the Meghna are not mutually exclusive of one another. That is, a family can live within 10 min of a market and also within 10 km of the Meghna. And although it is useful to compare individual strategies based on how close the families are to either a market or the Meghna, the results show that it may not just be proximity but rather distance that excludes families from engaging in a strategy. Families in the work-together strategy live farther away from a major market than families in any other strategy. They are most likely to live more than 10 min away from a market and live 63 min away on average. This distance is likely too far to make women’s selling a feasible occupation. Similarly, families in the split-year strategy live farther from the Meghna than those in any other strategy—an average of 9.42 km away. This distance may make fishing year-round an unprofitable venture and may limit the majority of fishing to the rainy season. Therefore, ecology determines which occupational opportunities are available to families, but also which opportunities are not available. Those that are not available primarily distinguish between split-year families, in which the division of labor is most divergent, and work-together families, in which the division of labor is most convergent.

### Alloparents Allow Mothers to Work away from Children

Alloparents across societies have been found to free mothers from the requirements of childcare, enabling them to spend more time away from home (e.g., Ivey 1993, 2000) and allowing them to return to work (e.g., Quinlan et al. 2003; Meehan 2009). Shodagor mothers in the split-year and leave-kids-home strategies are away from home for many hours a day and for at least half the year. This paper shows that split-year families are likely to have a child under the age of 5 and therefore have at least one child who needs constant monitoring. Mothers stay home as the primary caregiver for half of the year while fathers are working, and fathers stay home as the primary caregiver for the other half of the year while mothers are selling goods. It is not clear how these families benefit from alloparents, but split-year families have more alloparents than traditional or work-together families.

The same results show that leave-kids-home families are unlikely to have a child under the age of 5. This has two possible implications where alloparents are concerned. First, requirements for caregivers will be different: they will not be required to give constant attention to children who can swim, nor will they require the physical capabilities to save a child from the water. Second, these lowered requirements will result in an increased pool of available alloparents. Though caregivers’ responsibilities may be different, they still need to perform tasks such as feeding and providing general care for children over the age of 5 who can swim but who may not be able to cook or do other household tasks. Leave-kids-home families have statistically similar numbers of alloparents as split-year families, and more than work-together families.

Traditional and work-together families have the fewest number of available alloparents. For work-together families, this is consistent with Codding et al.’s (2011) suggestion that where division of labor converges on similar resources, fewer alloparents should be necessary. In this case, fewer alloparents are necessary because children are on the fishing boat with both parents. Alloparents are also less available for these families because most of the families who live nearby also employ the work-together strategy—which leaves very few potential caregivers in the bohor with whom parents could leave their children. In general, a convergent division of labor is associated with fewer alloparents being available, whereas families practicing a more divergent division of labor, in which mothers work away from their children, have more alloparents available.

### The Shodagor Division of Labor

The stage in a family’s domestic cycle, the specific aspects of their local ecology, and the availability of alloparents impact how Shodagor families divide labor within the household. The intersection of these three factors seems to explain why a family chooses a particular division of labor strategy and why the Shodagor families of Matlab have four distinct strategies they can employ.

The Shodagor ecology—homes surrounded by water throughout the year—imposes constraints on families with young children and necessitates that any caregiver be responsible and physically capable to mitigate the risk of child drowning. For families with young children who live close to a major market, women’s selling is only possible when such a caregiver is available. In the case of the split-year families, this caregiver is the children’s father, though alloparents are also available to help. Split-year families also live farther from the Meghna, which means fishing is profitable primarily during the rainy season. A combination of these constraints leads to men and women splitting the year between working and childcare, ensuring that one parent is providing for the family while the other keeps the children alive.

Families with young children who live relatively close to both the Meghna and a major market tend to employ the traditional strategy, with mother staying home year-round as the primary caregiver and father working outside the home year-round, fishing or working in the market or at a shop. The local ecology makes either option feasible. Lack of available alloparents likely motivates mothers to stay home; however, cultural factors may also lead some families to choose the traditional strategy. Many young women who do not work reported that their parents paid a higher dowry under the specific agreement that their daughters would not have to work after marriage. Parents may be doing this for a number of reasons. First, they could be concerned for their daughters’ safety, because selling goods can be risky (risks include transportation accidents, theft, or assault or harassment by village men of women traveling alone). Parents could also be concerned about their daughters’ reputations. Although others in Shodagor society do not hold women in low regard for working outside the home, this is not the case among village Bangladeshis, among whom purdah is common and women’s sexual reputations are closely guarded (Amin 1997). Some Shodagor parents may believe that protecting a daughter’s reputation among the larger village community may improve her status among both the Shodagor and the villagers. However, bivariate analyses show no evidence either that dowry was more likely to be paid for women in the traditional strategy (Table 1) or that a higher dowry was paid by these women’s parents (Table 2). More data is needed in order to flesh out this relationship.

For families who do not have a child under the age of 5 it is less important to have a parent at home with children all year, but these families are still constrained by the availability of alloparents as well as the local ecology. Leave-kids-home families live relatively close to both the Meghna and a major market, just as traditional families do. However, having older children and more alloparents available allows mothers and fathers to work away from home—and away from their children—year-round. Mothers often fish with their husbands during the rainy season when selling is difficult and sell goods during the dry season, while fathers typically fish all year.

Families who work together are not significantly different in age than families in any other strategy and are just as likely as all other families to have a child under the age of 5. The most relevant constraints for work-together families seem to be ecology and availability of alloparents. These families live close to the Meghna, which makes fishing profitable year-round. They also live farther from a major market than families in any other strategy, which limits women’s and men’s occupational choices to either fishing or staying home. Individuals who fish with their spouse or other members of the nuclear family do not have to split their catch or their income at the end of the day, which means that as long as there is no other, more profitable work available, spouses can earn the most money by working together. The lack of availability of alloparents necessitates children going along with their parents on the fishing boat.

For the Shodagor, having a child under the age of 5, travel time from a major market, distance from the Meghna River, and number of available alloparents interact to explain why labor is divided in particular ways. Specific ecological factors will differ based on the society, but when examining between-household differences in how labor is divided between husbands and wives, each of these variables is likely important in the model.

### Implications for the Study of the Sexual Division of Labor

Marlowe (2007) suggests that a deeper understanding of the sexual division of labor requires an examination of how labor is divided at the household level. He shows that a Hadza wife’s pregnancy or lactation status can affect the husband’s labor strategy (Marlowe 2003). Others show that the age of a husband or wife (Kaplan et al. 2000), age (Hurtado et al. 1985) or age distribution (Kramer 2004) of children, and presence of alloparents (Ivey 1993, 2000; Meehan 2009; Quinlan et al. 2003) impact individual subsistence practices. However, none of this research has looked at the impact of these circumstances on household subsistence strategies. Similarly, ecology impacts how men and women divide economic labor between societies (Codding et al. 2011; Jochim 1988; Marlowe 2007), but within-society or between-household differences have not been considered. Previous ecological models also have not included childcare tasks. The Shodagor offer an example of a society that has between-household differences in the division-of-labor strategies they employ. This paper shows the importance of including childcare in any household-level examination of the division of labor. The results also show that a family’s domestic cycle stage, local ecology, and availability of alloparents must be taken into consideration to fully comprehend the factors that distinguish between strategies.

Jochim’s (1988) and Marlowe’s (2007) ecological models regarding the sexual division of labor both focus on the resources found in a given ecology as a proxy for the subsistence opportunities that are available. The results from the Shodagor suggest that considering resources that are *not* available in a given ecology may be just as important to consider when attempting to understand why one society engages in particular subsistence tasks or pursues particular resources and not others. Formal application of optimal foraging theory would elucidate the point at which one strategy or occupation is not economical to pursue and should be excluded from consideration.

The Shodagor are an unusual culture in a number of ways, but the fact that some women and men engage in roles that are opposite of those found in nearly all other human societies is relevant to the study of how they divide labor. Shodagor women who sell goods are engaging in an occupation that is entirely incompatible with childcare. This is a behavior that is extremely unusual cross-culturally. Women who work with their husbands or pursue the same resources as their husbands engage in different tasks that are more compatible with childcare than the tasks men engage in (e.g., Hewlett 1992; Hurtado et al. 1985). Even most women in the modern West have occupations that are more likely to be compatible with breastfeeding and childcare than men’s (Bliege Bird and Codding 2015).

A little more than half of the women who sell goods also have children under the age of 5, which means that during the dry season when women are selling, they need an alternate caregiver for their children. In these cases, the father steps in and stays home for half of the year, opting to be the primary caregiver for his children rather than work outside the home. Hewlett (1992) reported that Aka fathers spend around 50% of their time in close proximity to their children, which is more than what has been reported for any other society (Marlowe 2000). And while Aka fathers’ care enabled mothers to engage in other tasks—just as alloparents have been shown to do in other societies—even Aka fathers are not the primary caregiver for their children all day, 6 months out of the year as this group of Shodagor fathers is. These families’ closeness to a major market provides selling opportunities for women, and their distance from the Meghna means that fishing is only reliably profitable during the rainy season. What is unclear, however, is why these fathers stay home even though these families have more available alloparents than families in other strategies. Perhaps they stay home because their opportunities to make money elsewhere are limited, and the benefit of fathers being better able to keep their children alive outweighs any added benefit that may come from additional income. Examination of time allocation data for fathers and alloparents during the dry season would be necessary to understand the duties performed by each and the benefits that come from those duties. Further analyses of the characteristics of fathers who stay home as well as the potential benefits to fathers, mothers, and children in these families is necessary to understand the implications on the sexual division of labor.

The purpose of this paper was to examine the differences between Shodagor families who engage in different strategies for dividing labor within the household. Three research questions were addressed regarding the impact of a family’s stage in the domestic cycle, local ecological variables, and the availability of alloparents. Each of these factors plays a role individually in explaining why Shodagor men and women engage in different occupations, but the intersection of all three factors best explains why a family chooses one division of labor strategy over the other three practiced by the Matlab Shodagor. Most models of the division of labor examine the effect of one or two variables on how men and women as broad groups divide labor within or across societies. These models do not typically explore the intersecting effects of multiple variables, nor do they look at the division of labor at the level of the household. These steps would be useful in future attempts to understand how and why labor is divided between men and women in different cultures.

## References

[CR1] Ahmed N, Owen JE (1962). The Laua community. Sociology in East Pakistan.

[CR2] Amin S (1997). The poverty-purdah trap in rural Bangladesh: implications for women’s roles in the family. Development and Change.

[CR3] Amin S (1998). Family structure and change in rural Bangladesh. Population Studies.

[CR4] Amin S, Cain M, Jones GW, Douglas RM, Caldwell JC, D’Souza RM (1997). The rise of dowry in Bangladesh. The continuing demographic transition.

[CR5] Aziz KMA (1979). Kinship in Bangladesh.

[CR6] Bangladesh Bureau of Statistics (2010). Area, population, household, and household characteristics. The Statistical Yearbook of Bangladesh – 2010*.* www.bbs.gov.bd. Accessed 15 May 2016.

[CR7] Becker GS (1985). Human capital, effort, and the sexual division of labor. Journal of Labor Economics.

[CR8] Bliege Bird R (1999). Cooperation and conflict: the behavioral ecology of the sexual division of labor. Evolutionary Anthropology.

[CR9] Bliege Bird R (2007). Fishing and the sexual division of labor among the Meriam. American Anthropologist.

[CR10] Bliege Bird R, Bird DW (1997). Delayed reciprocity and tolerated theft: the behavioral ecology of food sharing strategies. Current Anthropology.

[CR11] Bliege Bird R, Codding BF, Scott R, Kosslyn S (2015). The sexual division(s) of labor. Emerging trends in the social and behavioral sciences.

[CR12] Bliege Bird R, Smith EA, Bird DW (2001). The hunting handicap: costly signaling in male foraging strategies. Behavioral Ecology and Sociobiology.

[CR13] Brown JK (1970). A note on the division of labor by sex. American Anthropologist.

[CR14] Bureau of Labor Statistics. (2014). *Labor force statistics from the current population survey.* http://www.bls.gov/cps/cpsaat11.htm. Accessed 14 Jan 2016

[CR15] Codding BF, Bliege Bird R, Bird DW (2011). Provisioning offspring and others: risk–energy trade-offs and gender differences in hunter-gatherer foraging strategies. Proceedings of the Royal Society B: Biological Sciences.

[CR16] Dahlberg F (1981). Woman the gatherer.

[CR17] Ember CR (1975). Residential variation among hunter-gatherers. Cross-Cultural Research.

[CR18] Fortes M, Goody J (1958). Introduction. The developmental cycle in domestic groups.

[CR19] Geary DC (2000). Evolution and proximate expression of human paternal investment. Psychological Bulletin.

[CR20] Goodman, M. J., Griffin, P. B., Estioko-Griffin, A. A., Goodman, M. J., et al. (1985). Menarche, pregnancy, birth spacing and menopause among the Agta women foragers of Cagayan Province, Luzon, The Philippines. *Annals of Human Biology, 12*(2), 169–177. 10.1080/03014468500007661 3985568

[CR21] Gurven M, Hill K (2009). Why do men hunt? A reevaluation of “man the hunter” and the sexual division of labor. Current Anthropology.

[CR22] Hames RB (1979). A comparison of the efficiencies of the shotgun and the bow in neotropical forest hunting. Human Ecology.

[CR23] Hames, R. B. (1988). The allocation of parental care among the Ye’kwana. In L. Betzig, M. Borgerhoff Mulder, and P. Turke (Eds.) *Human reproductive behavior* (pp. 237–251). Cambridge: Cambridge University Press.

[CR24] Hames RB, Hewlett B (1992). Variation in paternal care among the Yanomamo. Father-child relations: cultural and biosocial contexts.

[CR25] Hames RB, Draper P (2004). Women’s work, child care, and helpers-at-the-nest in a hunter-gatherer society. Human Nature.

[CR26] Harkness S, Super CM, Hewlett BS (1992). The cultural foundations of fathers’ roles: evidence from Kenya and the United States. Father-child relations: cultural and biosocial context.

[CR27] Hawkes K, Cashdan EA (1990). Why do men hunt? Some benefits for risky strategies. Risk and uncertainty in tribal and peasant economies.

[CR28] Hawkes K (1991). Showing off: tests of an hypothesis about men’s foraging goals. Ethology and Sociobiology.

[CR29] Hewlett BS (1991). Intimate fathers: the nature and context of aka pygmy paternal infant care.

[CR30] Hewlett BS, Hewlett BS (1992). Husband-wife reciprocity and the father-infant relationship among Aka pygmies. Father-child relations: cultural and biosocial context.

[CR31] Hewlett BS, Lamb ME (2005). Hunter-gatherer childhoods: evolutionary, developmental, and cultural perspectives.

[CR32] Hill K (1988). Macronutrient modifications of optimal foraging theory: an approach using indifference curves applied to some modern foragers. Human Ecology.

[CR33] Hill K, Kaplan H (1993). Why do male foragers hunt and share food?. Current Anthropology.

[CR34] Hofer T, Messerli B (1997). Floods in Bangladesh: history, dynamics, and rethinking the role of the Himalayas.

[CR35] Hrdy S (1992). Fitness tradeoffs in the history and evolution of delegated mothering with special reference to wet-nursing. Ethology and Sociobiology.

[CR36] Hurtado AM, Hawkes K, Hill K, Kaplan H (1985). Female subsistence strategies among Ache hunter-gatherers of eastern Paraguay. Human Ecology.

[CR37] Hurtado AM, Hill K, Hurtado I, Kaplan H (1992). Trade-offs between female food acquisition and child care among Hiwi and Ache foragers. Human Nature.

[CR38] International Centre for Diarrheal Disease Research, Bangladesh (ICDDRB). (2014). *Health and demographic surveillance system–Matlab*. Bangladesh: Dhaka.

[CR39] Ivanoff J, Cholmeley FN, Ivanoff P (1997). Moken: sea-gypsies of the Andaman Sea, post-war chronicles.

[CR40] Ivey PK (1993). Life-history theory perspectives on allocaretaking strategies among Efe foragers of the Ituri Forest of Zaire.

[CR41] Ivey PK (2000). Cooperative reproduction in Ituri Forest hunter-gatherers: who cares for Efe infants?. Current Anthropology.

[CR42] Jochim MA (1988). Optimal foraging and the division of labor. American Anthropologist.

[CR43] Kaplan H, Hill K, Hurtado AM, Cashdan E (1990). Risk, foraging and food sharing among the ache. Risk and uncertainty in tribal and peasant economies.

[CR44] Kaplan H, Hill K, Lancaster J, Hurtado AM (2000). A theory of human life history evolution: diet, intelligence, and longevity. Evolutionary Anthropology.

[CR45] Kaplan HS, Hooper PL, Gurven M (2009). The evolutionary and ecological roots of human social organization. Philosophical Transactions of the Royal Society, B.

[CR46] Kelly R (1995). The foraging spectrum: diversity in hunter-gatherer lifeways.

[CR47] Konner M, Hewlett BS, Lamb ME (2005). Hunter-gatherer infancy and childhood: the !Kung and others. Hunter-gatherer childhoods: evolutionary, developmental, and cultural perspectives.

[CR48] Kramer K (2004). Reconsidering the cost of childbearing: the timing of children’s helping behavior across the life cycle of Maya families. Research in Economic Anthropology.

[CR49] Lamb ME, Pleck JH, Chamov EL, Levine JA (1985). Paternal behavior in humans. American Zoologist.

[CR50] Lancaster, J. B. & Lancaster, C. S. (1983) Parental investment: the hominid adaptation. In D. Ortner, (Ed.),. *How humans adapt: A biocultural odyssey,* pp. 33–66. Proceedings of the Seventh International Smithsonian Symposium. Washington DC: Smithsonian Institution.

[CR51] Lee RB, Lee RB, DeVore I (1968). What hunters do for a living, or how to make out on scarce resources. Man the hunter.

[CR52] Lee RB (1979). The !Kung San: men, women, and work in a foraging community.

[CR53] Lee R, DeVore I (1968). Man the hunter.

[CR54] Lovejoy CO (1981). The origin of man. Science.

[CR55] Marlowe F (1999). Male care and mating effort among Hadza foragers. Behavioral Ecology and Sociobiology.

[CR56] Marlowe F (1999). Showoffs or providers? The parenting effort of Hadza men. Evolution and Human Behavior.

[CR57] Marlowe F (2000). Paternal investment and the human mating system. Behavioural Processes.

[CR58] Marlowe, F. (2001). Male contribution to diet and female reproductive success among foragers. *Current Anthropology, 42*, 755–760.

[CR59] Marlowe, F. (2003). A critical period for provisioning by Hadza men: Implications for pair bonding. *Evolution and Human Behavior, 24*, 217–229.

[CR60] Marlowe F (2007). Hunting and gathering: the human sexual division of foraging labor. Cross-Cultural Research.

[CR61] Meehan CL (2009). Maternal time allocation in two cooperative childrearing societies. Human Nature.

[CR62] Munroe RL, Munroe RH, Hewlett BS (1992). Fathers in children’s environments: a four culture study. Father-child relations: cultural and biosocial context.

[CR63] Novak JJ (1993). Bangladesh: reflections on the water.

[CR64] O’Connell JF, Hawkes K, Winterhalder B, Smith EA (1981). Alyawara plant use and optimal foraging theory. Hunter-gatherer foraging strategies: ethnographic and archaeological analyses.

[CR65] Pate D (1986). The effects of drought on Ngatatjara plant use: an evaluation of optimal foraging theory. Human Ecology.

[CR66] Quinlan RJ, Quinlan MB (2008). Human lactation, pair-bonds, and alloparents. Human Nature.

[CR67] Quinlan RJ, Quinlan MB, Flinn MV (2003). Parental investment and age at weaning in a Caribbean village. Evolution and Human Behavior.

[CR68] Sather C (1997). The Bajau Laut: adaptation, history, and fate in a maritime fishing society of south-eastern Sabah.

[CR69] Sear R, Mace R (2008). Who keeps children alive? A review of the effects of kin on child survival. Evolution and Human Behavior.

[CR70] Starkweather KE (2016). Merchant mothers and fishermen fathers: parental investment and subsistence work among the boat-dwelling Shodagor of rural Bangladesh.

[CR71] Stephens DW, Krebs JR (1986). Foraging theory.

[CR72] Trivers R, Campbell B (1972). Parental investment and sexual selection. Sexual selection and the descent of man 1871–1971.

[CR73] UNICEF (2009). Bangladesh health and injury survey.

[CR74] von Rueden C (2011). The acquisition of social status by males in small-scale human societies.

[CR75] Winking J, Gurven M, Kaplan H, Stieglitz J (2009). The goals of direct paternal care among a South Amerindian population. American Journal of Physical Anthropology.

[CR76] Winterhalder B, Winterhalder B, Smith EA (1981). Foraging strategies in the boreal environment: an analysis of Cree hunting and gathering. Hunter-gatherer foraging strategies: ethnographic and archaeological analyses.

