# Erratum to: Shodagor Family Strategies: Balancing Work and Family on the Water

**DOI:** 10.1007/s12110-017-9295-x

**Published:** 2017-05-30

**Authors:** Kathrine E. Starkweather

**Affiliations:** 1 University of Missouri, 1400 University Place, Columbia, MO 65211 USA; 2 Max Planck Institute for Evolutionary Anthropology, Deutscher Platz 6, 04103 Leipzig, Germany

**Erratum to: Hum Nat**

**DOI** **10.1007/s12110–017-9285-z**

The article Shodagor Family Strategies: Balancing Work and Family on the Water, written by Kathrine E. Starkweather, was originally published electronically on the publisher’s internet portal (currently SpringerLink) on March 11, 2017 without open access.

With the author’s decision to opt for Open Choice the copyright of the article changed in May 2017 to © The Author(s) 2017 and the article is forthwith distributed under the terms of the Creative Commons Attribution 4.0 International License (http://creativecommons.org/licenses/by/4.0/), which permits use, duplication, adaptation, distribution and reproduction in any medium or format, as long as you give appropriate credit to the original author(s) and the source, provide a link to the Creative Commons license and indicate if changes were made.

The original article has been corrected.

